# Supporting treatment adherence for resilience and thriving (START): protocol for a mHealth randomized controlled trial

**DOI:** 10.1186/s12889-024-19745-7

**Published:** 2024-08-29

**Authors:** Leah Davis-Ewart, Lindsay Atkins, Delaram Ghanooni, José E. Diaz, Chika C. Chuku, Raymond Balise, Britt A. DeVries, Michael Miller-Perusse, Donovan Ackley III, Judith T. Moskowitz, Kathryn McCollister, Erminia Fardone, Sabina Hirshfield, Keith J. Horvath, Adam W. Carrico

**Affiliations:** 1 Department of Disease Prevention and Health Promotion, Robert Stempel College of Public Health & Social Work, Florida International University, 11200 S.W. 8th Street, AHC5, #407, Miami, Fl 33199 USA; 2 Department of Psychology, San Diego State University, San Diego, CA USA; 3 Department of Medicine, State University of New York Downstate Health Sciences University, New York, NY USA; 4 Department of Public Health Sciences, University of Miami Miller School of Medicine, Miami, FL USA; 5 Department of Medical Social Sciences, Northwestern University School of Medicine, Chicago, IL USA

**Keywords:** HIV, mHealth, Stimulant use, Men who have sex with men, Dried blood spots, Viral load

## Abstract

**Background:**

Although behavioral interventions show some promise for reducing stimulant use and achieving durable viral suppression in sexual minority men (SMM) with HIV, scalable mHealth applications are needed to optimize their reach and cost-effectiveness.

**Methods:**

Supporting Treatment Adherence for Resilience and Thriving (START) is a randomized controlled trial (RCT) testing the efficacy and cost-effectiveness of a mHealth application that integrates evidence-based positive affect regulation skills with self-monitoring of adherence and mood. The primary outcome is detectable HIV viral load (i.e., > 300 copies/mL) from self-collected dried blood spot (DBS) specimens at 6 months. Secondary outcomes include detectable DBS viral load at 12 months, self-reported stimulant use severity, anti-retroviral therapy (ART) adherence, and positive affect over 12 months. A national sample of up to 250 SMM with HIV who screen positive for stimulant use disorder and reporting suboptimal ART adherence is being recruited via social networking applications through April of 2024. After providing informed consent, participants complete a run-in period (i.e., waiting period) including two baseline assessments with self-report measures and a self-collected DBS sample. Those who complete the run-in period are randomized to either the START mHealth application or access to a website with referrals to HIV care and substance use disorder treatment resources. Participants provide DBS samples at baseline, 6, and 12 months to measure HIV viral load as well as complete self-report measures for secondary outcomes at quarterly follow-up assessments over 12 months.

**Discussion:**

To date, we have paid $117,500 to advertise START on social networking applications and reached 1,970 eligible participants ($59.77 per eligible participant). Although we identified this large national sample of potentially eligible SMM with HIV who screen positive for a stimulant use disorder and report suboptimal ART adherence, only one-in-four have enrolled in the RCT. The run-in period has proven to be crucial for maintaining scientific rigor and reproducibility of this RCT, such that only half of consented participants complete the required study enrollment activities and attended a randomization visit. Taken together, findings will guide adequate resource allocation to achieve randomization targets in future mHealth research SMM with HIV who use stimulants.

**Trial Registration:**

This protocol was registered on clinicaltrials.gov (NCT05140876) on December 2, 2021.

**Supplementary Information:**

The online version contains supplementary material available at 10.1186/s12889-024-19745-7.

## Background

The prevalence of stimulant use, including methamphetamine (meth), is substantially elevated among sexual minority men (SMM) in the United States [1–8]. Recent data show that SMM are more than twice as likely to report recent meth use compared to heterosexual men [9, 10]. Despite successful deployment of public health interventions that specifically targeted meth use in SMM [11–13], the meth use epidemic has surged in recent years [14]. For example, meth use doubled nationwide 2011–2019, with similar evidence emerging across major Ending the HIV Epidemic (EHE) jurisdictions [3, 15, 16]. There is also increasing recognition that meth and other stimulant use is prevalent in ethnic minority SMM, a population in which HIV incidence is the highest [15]. Findings from our team indicate that 20% of young Black SMM in Texas reported stimulant use in the past 2 months [17]. Because meth and other stimulant use have consistently been identified as potent drivers of the HIV/AIDS epidemic [18–20], there is a clear and present need to bolster efforts to address the intertwining epidemics of stimulant use and HIV among SMM.

Stimulant use undermines the clinical and public health benefits of HIV Treatment as Prevention (TasP), one of the primary biomedical tools for controlling the HIV epidemic among SMM [21–23]. People living with HIV who achieve and maintain viral suppression (i.e., < 200 copies/mL) have better health outcomes and virtually eliminate risk of HIV transmission to sexual partners [24–26]. Among SMM with HIV, stimulant use is associated with greater difficulties navigating the HIV care continuum, including a lower likelihood of engagement in HIV care [27], lower adherence to antiretroviral therapy (ART) [28–30], and greater odds of unsuppressed viral load (VL) [27, 28, 31, 32]. Even in the era of TasP, we observed slower rates of viral suppression among this population [28] and our recent findings demonstrate that SMM with HIV who use stimulants display alarmingly high risk of viral rebound [28]. The difficulties this population faces achieving and maintaining viral suppression potentiates hastened clinical HIV progression [33–35] as well as amplified risk for onward HIV transmission [30, 36, 37]. Expanded efforts are needed to increase rates of durable viral suppression among SMM with HIV who use stimulants, a high priority population that experiences greater rates of HIV-associated comorbidities and are at increased risk for engaging in HIV transmission risk behavior [30, 36–40]. Taken together, there is an urgent need for scalable behavioral interventions to assist SMM with HIV who use stimulants with achieving and maintaining viral suppression in the era of TasP.

Randomized controlled trials (RCTs) support the efficacy of intensive behavioral interventions such as cognitive-behavioral therapy, motivational interviewing, and contingency management (CM), for reducing substance use and sexual risk taking among SMM who use stimulants [18, 41, 42]. Our team demonstrated the efficacy of a 5-session individually delivered positive affect regulation intervention (ARTEMIS) delivered during CM for stimulant abstinence with 110 SMM with HIV who use methamphetamine. Informed by the revised Stress and Coping Theory [43], the ARTEMIS positive affect intervention targets fundamental neurobehavioral factors such as withdrawal and anhedonia that are key features of stimulant use disorders [44, 45]. Men randomized to receive the ARTEMIS intervention reported decreases in meth craving and stimulant use during the 3-month CM intervention period [46]. Furthermore, men randomized to receive the ARTEMIS intervention reported increases in positive affect as well as decreases in the frequency of stimulant use at six and 12 months that paralleled durable and clinically meaningful reductions in VL over 15 months. To date, ARTEMIS is the only behavioral intervention model that has demonstrated durable efficacy for achieving clinically meaningful reductions in VL in people with HIV who use substances [47, 48]. Although the ARTEMIS intervention model is efficacious, it is also resource intensive. Novel approaches are needed to surmount enduring barriers to scalability of the ARTEMIS positive affect regulation skills to reach the larger population of SMM with HIV who use stimulants, particularly those residing outside of major urban centers.

Establishing the efficacy of mHealth applications for SMM with HIV who use stimulants is crucial to reach the EHE goals [49]. Both mHealth (i.e., interventions delivered via mobile devices) and eHealth (i.e., interventions delivered or enhanced via Internet and related technologies) for ART adherence have proliferated [50–52] due to the widespread adoption of technology [53], the ability to reach a broad audience, rapid scalability, consistent and “real-time” delivery, and relatively low implementation costs [54, 55]. For these reasons, mHealth and eHealth technologies may overcome the limitations of in-person and clinic-based interventions [56].

A review of mHealth/eHealth interventions [54] highlights that the majority of effective interventions targeting substance use among people with HIV have also sought to optimize ART adherence or engagement in HIV care [56–58]. However, many mHealth/eHealth interventions designed for substance use were not tailored to SMM. SMM with HIV who use substances experience multiple, overlapping sources of stigma, discrimination, and prejudice related to being a sexual minority, substance use, and HIV. There is a clear need for mHealth/eHealth interventions to address co-occurring stimulant use and HIV among SMM. A review highlighted only seven mHealth applications targeting ART adherence that have been developed for SMM to date, and none specifically targeted SMM with HIV who use substances [52]. Our team completed a pilot RCT of a mHealth ART adherence application (APP+) with 90 ART-treated SMM with HIV who use stimulants. Grounded in the Information-Motivation-Behavioral (IMB) skills model [59], participants receiving APP + reported greater ART adherence during the 4-month intervention period, although these gains were not maintained at month 6. Participants randomized to APP + also reported greater reductions in stimulant use at 4 and 6 months.

**Fig. 1:**
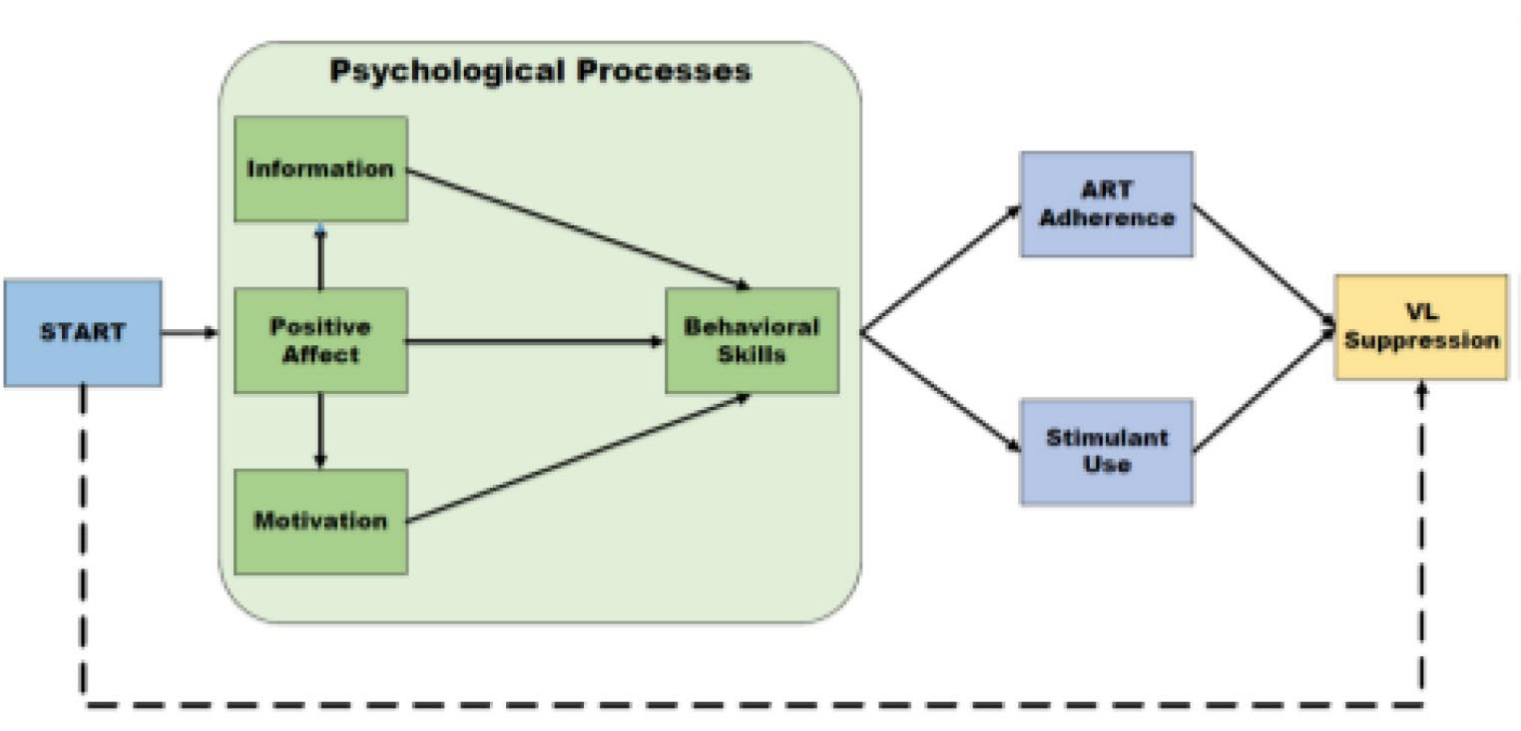
START study conceptual model

The IMB model provides information relevant to co-occurring stimulant use and HIV, motivational enhancements, and behavioral skills (e.g., self-monitoring) to improve ART adherence. However, there is clear recognition that prevalent psychiatric comorbidities such as mental health and substance use disorders could mitigate many of the benefits of IMB processes for improving ART adherence [60], which underscores the potential benefits of the ARTEMIS positive affect regulation skills. Revised Stress and Coping Theory proposes that positive affect has unique adaptive consequences amid chronic stress such as living with HIV/AIDS [43, 61–63]. Positive affect is theorized to be crucial for reinvigorating and sustaining coping efforts during chronic stress to improve psychological adjustment and support health behavior change. This is supported in part by prior research that positive affect is associated with HIV-related health behavior change such as engagement in HIV care and ART adherence as well as decreased stimulant use [61–63]. Guided by our integrative conceptual model (see Fig. 1), we hypothesize that there will be synergistic benefits to integrating the ARTEMIS positive affect regulation skills and APP + IMB skills into a single mHealth application, called START (Supporting Treatment Adherence for Resilience and Thriving).

START leverages at-home dried blood spot (DBS) collection to monitor VL, which is common in trials occurring outside of clinical settings due to its feasibility and acceptability [64]. DBS sampling has been used in non-clinical settings for quantifying VL to identify acute and undiagnosed HIV infections [65, 66]. However, DBS collection in non-clinical settings for VL quantification in people with HIV has largely been unexplored until recently. HemaSpot™ HD and HF devices are designed to collect larger samples for a variety of assays [67, 68]. These devices were developed to address technical issues including effects of exposure to light, temperature, and humidity associated with using traditional DBS filter cards. In addition, these devices include a protective plastic cartridge which minimizes the risk of contamination. Our prior research using HemaSpot™ kits to collect DBS for lab-quantified VL enrolled 554 SMM with HIV online, which resulted in a 75% return rate [69]. Over half of the men (53%) had a detectable VL lab result (median: 3,508 copies/ mL; range: 851–1,202,265 copies/mL); among men with a detectable VL, 84% self-reported their VL status as undetectable at their last clinical visit. These discrepancies have significant ramifications for amplified risk of HIV transmission, as inaccurate perception of one’s VL status may influence engagement in condomless sex with uninfected sexual partners.

This protocol describes an ongoing RCT testing the efficacy and cost-effectiveness of the START mHealth application, which combines the evidence-based ARTEMIS and APP + interventions. The primary outcome is detectable HIV viral load (i.e., > 300 copies/mL) from self-collected DBS specimens at 6 months. Secondary outcomes include detectable DBS viral load at 12 months as well as self-reported stimulant use severity, ART adherence, and positive affect over 12 months. Our team is also assessing the potential cost-effectiveness of the START mHealth application, including net savings with respect to averted healthcare utilization if it ultimately proves to be efficacious in reducing VL.

## Methods

In contrast to many prior RCTs of behavioral interventions for SMM with HIV who use stimulants, this RCT is fully remote such that participants complete screening, enrollment, and all RCT activities from home. Potentially eligible participants are identified in an online screener and contacted by the study team to provide informed consent for the RCT procedures. After providing informed consent, participants complete a run-in period (i.e., waiting period) that includes two baseline assessments with self-report measures and a self-collected DBS sample. Those who complete the run-in period attend a separate onboarding visit where they are randomized to receive either the START mHealth application or access to a website with referrals to HIV care and substance use disorder treatment resources. Participants provide DBS samples at baseline, 6 and 12 months to measure HIV viral load as well as complete self-report measures for secondary outcomes at quarterly follow-up assessments over 12 months. Participants receive up to $500 in incentives using gift card codes that can be redeemed at multiple online retailers for completing all trial activities over the 12-month period. The University of Miami serves as the single IRB for this RCT, and reliance agreements were executed with San Diego State University, the State University of New York – Downstate, and Florida International University. The University of California, Los Angeles Data Safety and Monitoring Board in Addiction Medicine oversees the execution of this RCT via annual reports.

### Recruitment and screening

Participants are recruited online from social networking websites and applications, consent to contact databases, and a national advertisement in POZ magazine. National advertising campaigns for START using male-for-male social networking sites began in June 2022 at a cost of $117,500 to date. We utilize national consent-to-contact databases from other completed and ongoing national research studies conducted with SMM. Potential participants who click on a study banner ad or link within a recruitment email will access the online screening survey to determine eligibility. Eligible participants will include those reporting: (a) assigned male sex at birth; (b) identifies as male; (c) age 18 or older; (d) ability to read and speak English; (e) US residency; (f) reports ever having had anal sex with a man; (g) HIV diagnosis for 3 or more months and currently taking ART; (h) reports detectable VL during the past year or no viral load test in the past 10 + months or < 90% adherence to ART; (i) a positive screen for moderate-to-severe stimulant use disorder in the past three months with an abbreviated version of the NIDA-modified Alcohol, Smoking, and Substance Involvement Screening Test (ASSIST) [70]; (j) Has an iPhone or Android smartphone; and (k) is willing to participate in an mHealth intervention. Men are immediately informed if they are eligible to participate in the START RCT, but all participants are asked to provide their contact information if they would like to be in a consent to contact database for future projects.

### Enrollment and run-in period

Based on eligibility via the online screener, participants are added to a list managed using REDCap [71, 72] and contacted by study staff to schedule an enrollment visit. Study staff reach out via Textel [73] or email, depending on the participant’s preferred method of contact, to schedule a visit. A Zoom [74] meeting is scheduled with study staff for the informed consent process, which includes a discussion of the procedures for the RCT. Consent or lack thereof is documented in the electronic database. At the enrollment visit, participants are also asked to upload a photo of their HIV medication bottle via a secure REDCap link as documentation that they are currently taking ART.

After completing the enrollment visit, participants are asked to complete a brief run-in period prior to randomization. This includes completing two baseline assessments with self-report measures for the RCT and providing a viable DBS specimen for VL testing. This ensures that all randomized participants can adhere to the RCT procedures and have provided a sufficient DBS specimen to measure VL at baseline. This is particularly important given our prior findings that stimulant-using SMM with HIV experience greater difficulties completing DBS self-collection to measure VL than SMM with HIV not using stimulants [75].

All participants receive a HemaSpot™ HD or HF DBS kit for specimen collection, which they are instructed to return by mail to the University of Miami laboratory. The DBS collection kit sent via the United States Postal Service contains the HemaSpot™ HD or HF device, 2 alcohol prep pads, 2 lancets, 1 gauze pad, and one adhesive bandages. The kit includes a card with instructions on how to collect the DBS sample and a QR code to an instructional video. After completing the DBS specimen collection, participants are asked to apply the label with their participant ID and timepoint before placing it in a biohazard bag for shipment. The DBS specimen is then placed in prepaid return envelope addressed to the University of Miami laboratory with a sheet which includes the participant ID. Once the kit arrives at the University of Miami laboratory, it undergoes a thorough validation process. If it is determined to be valid, it is carefully labeled, sealed inside a storage bag along with desiccant pellets and a humidity indicator card. Subsequently, the sealed kit is stored in a -20-degree freezer within a dedicated container. Once the container reaches its capacity, it is transferred to a -80-degree freezer for long-term storage.

Samples stored in the − 80-degree freezer are periodically dispatched for VL analysis to Dr. Rami Kantor’s laboratory at Brown University. The HIV viral load at Dr. Kantor’s laboratory is determined using the Abbott m2000 RealTime HIV-1 DBS quantitative assay (Abbott Molecular Inc., Des Plaines, IL). This is an in vitro reverse transcription-polymerase chain reaction assay for the quantitation of HIV-1 RNA from HIV-1 infected individuals. The viral load testing is done on Abbott m2000 platform that includes the m2000sp instrument for automated extraction of RNA and the m2000rt instrument for real-time PCR analysis. It is important to note that the assay targets the pol integrase region of the HIV-1 genome and has a limit of detection of 831 copies/mL [76].

### Randomization and onboarding

Men who complete the run-in period are asked to attend a separately scheduled onboarding visit with study staff via Zoom where they are randomized using a computer-generated algorithm developed by the study statistician (Dr. Balise) for block randomization in randomly permuted blocks of 2, 4, and 6 to guard against subversion. Randomization will be stratified by whether participants are actively seeking formal substance use disorder treatment to achieve balance on this important possible moderator. Treatment and non-treatment seeking men may likely have different intervention needs, which may differentially impact their experience with the START mHealth application and outcomes. Following randomization, participants are provided with a brief overview of the START mHealth application or informational control website that includes referrals to HIV care and substance use disorder treatment resources.

### START mHealth application

Those randomized to receive the app have an account created by the START staff member, are asked to download the app, and are given a START app tutorial during their onboarding visit. Participants are instructed to log into the app and are guided through the Check-Ins, Welcome Videos, My Settings page, Crisis Resources, Skills, Self-Care Check List and other key components (Table 1). Participants are asked to begin the first lesson, Positive Events and Gratitude, and make their way through each lesson on the app as it unlocks. If they have any questions, they are encouraged to reach out to START staff via text or email. Additionally, they are asked to engage with the app several times per week for six months.

**Table 1:** START intervention app components and features

Features	Description	Format	Activities
Check-In	Check in on mood, adherence, using drugs/alcohol	Text, graphics	Prompts participant to choose current mood, report if they took their HIV medications yet, and report if they are currently using drugs/alcohol.
Welcome	Introduction to the START app and investigators, self-care, and information about stimulant use and HIV	Text, graphics, video	Choose self-care strategies to implement when stressed
Positive Events and Gratitude	Noticing positive events, developing gratitude	Text, graphics, video	● Write down 3 good things that happened today ● Write down 1 + thing that the participant is grateful for ● Meditation – Breath retraining
Mindfulness and Self-Compassion	Informal mindfulness, self-compassion	Text, graphics, video	● Write down 1 + things that participant wants to be mindful of ● Choose the self-compassionate statement in 4 multiple choice scenarios; practice using self-compassion skills on personal situations ● Meditation – Mindful breathing
Reappraisal and Coping	Learning positive reappraisal and coping skills	Text, graphics, video	● Choose the positive reappraisal statement in 3 multiple choice scenarios; practice using positive reappraisals on personal situations Meditation – Mountain meditation
Values, Strengths, and Goals	Taking inventory of values and strengths; applying values and strengths to setting goals	Text, graphics, video	● Practice on 2 scenarios to set an attainable goal, ordering steps to achieve goal, and attaching values and strengths ● Set own attainable goal, setting steps, and attaching values and strengths, track during check in. ● Meditation- Mindful meditation
Acts of Kindness	Learning how to enact acts of kindness	Text, graphics, video	● Activity to help create 1 + acts of kindness and track it ● Meditation – Loving kindness meditation
My Skills	Display of Skills with badges representing time completed, direct access to skills practice, and direct access to meditations	Text, graphics	
My Timeline	Timeline of activities completed by month and day	Text, graphics	
My Trends	Summary of Check in data		
My Self Care	List of self-care activities that can be checked off and edited	Text	
Crisis Resources	List of crisis, LGBTQ, mental health and substance use, and HIV and health resources	Text, graphics	
My settings	Change PIN, Contact us, FAQ, set and update medication reminder, set and update check-in reminder	Text	

### Informational control website

START participants randomized into the control arm of the study will have access to the START Study website. The goal of the website is to provide participants with resources in four distinct categories: crisis resources, LGBTQ resources, mental health and substance use resources, and HIV and health resources. Resources include website access, phone numbers to call, and lines to text depending on contact preference.

Crisis Resources include 988 Suicide and Crisis Lifeline, Crisis Text Line, THRIVE Lifeline, and The Trevor Project Lifeline. These crisis resources offer access to local crisis centers that provide free and confidential emotional support to people in suicidal crisis or emotional distress 24 h a day, 7 days a week.

LGBTQ Resources include LGBT National Help Center, LGBT Near Me, CenterLink, Centers for Disease Control and Prevention, and TrevorSpace. These resources provide free and confidential peer support, information, and referrals to local resources.

Mental Health and Substance Use Resources include National Institute of Mental Health, The National Alliance on Mental Illness, Mental Health America, Substance Abuse and Mental Health Services Administration, and North American Syringe Exchange Network. These resources provide information about mental health for LGBTQ people, important risk factors, and finding help for mental health and substance use.

HIV and Health Resources include Health Resources and Services Administration, The Body, Greater than AIDS, Be in the KNOW, and POZ. These resources provide information about locating local HIV providers, HIV/AIDS related information and support, and access to the latest information, news, and community forums regarding the needs of people living with and affected by HIV/AIDS. Participants are encouraged to visit the control website as often or as little as they would like and to reach out to any of the resources when needed.

### Follow-up study procedures and retention

All participants will receive an email with a link to the 3-, 6-, 9-, and 12-month follow-up surveys post randomization. The behavioral surveys will take approximately 30–45 min to complete. They also receive DBS kits at 6- and 12-months.

#### Study Retention

Study participants provide email and text contact information following the formal consent process. Study staff are assigned specific participants with whom to follow up for each study activity, including surveys, DBS home test kits, and randomized treatment or control group assignment. Staff engage participants one-on-one with various text and / or email reminders when participants have not responded to or completed survey invitations, or when they have not returned a DBS kit. Engagement contacts are recorded in each participant record in the study database (REDCap) and follow up is continued for at least three attempts for surveys and at least eight attempts for DBS home test kits. Participants are paid 2–3 business days after completion of each study activity.

***Procedures to maximize engagement in the app***. START Study engagement strategies are outlined in the standard operating procedures (SOP) given to all study staff. Study staff are also trained on participant engagement through one-on-one coaching by the project coordinator.

1. Study participants are contacted periodically after assignment to the treatment or control group to support their use of the treatment app or the control website. Coordinators check in with treatment group participants within a week after their onboarding visit to ensure the app is working correctly. Additionally, participants will receive a follow up message with resources after the randomization visit with a link to the control website or a link to a video walkthrough of the app. Specifically, study coordinators check in ~ 5 day after enrollment, 7–14 days of inactivity with app, and any participants 30 + days inactive.
2. Additional START User Engagement Procedures have been implemented and added to START Study SOP. Engagement and retention seem to be improving as we apply these new procedures.
  2.1. Weekly analysis and reporting on Treatment Group activity: Study staff assigned to engagement reporting reviews daily email from intervention app developer, Radiant, listing study participants assigned to the treatment group who have not opened the intervention (app) for 15 days or more. Using a coordinator account, staff then accesses backend user reports on the app developer’s website to review inactive participants’ check-in data against the dates they opened the app to ensure accuracy. If a discrepancy is noted in these reports, staff reaches out to the app developer to investigate potential tech issues.
  2.2. Weekly Coordinator Reports and participant outreach: Study staff assigned to engagement reporting reviews all backend user reports on the app developer’s website to track activity of each participant assigned to the Treatment Group in each activity and each module, number of reminders set, and number of videos watched. Each study coordinator receives a full weekly report on all activities of those who have been inactive 15 days or more, with recommendations for outreach suggesting activities the participant has not yet tried or any emerging or recurring patterns that may be useful to the coordinator. Coordinators reach out to participants on this weekly report by text or email to ask if the participant is having any trouble with the app and to suggest news ways to engage. When participants reply with tech issues, such as a forgotten PIN number or new phone, study coordinators help the user resolve the issue and re-engage with the study intervention.
  2.3. Monthly Engagement Report: The same procedures that are done weekly for Inactive Users are applied to all participants assigned to the Treatment Group once a month, including both active and inactive. Discrepancies are reported to the app developer to identify potential reporting errors or tech issues, and the project coordinator and engagement staff meet with the developer on a regular half-hour weekly video conference call to investigate and address these issues. Coordinators are notified of any issues involving their participants that have been brought to the app developer. Each month, study coordinators are sent a full report on the check-in activity and time in app (times opened) for all their participants, with more detailed notes on individual engagement to personalize their interactions with participants.
  2.4. Month-to-month comparison for optimization: The activities of all participants in the treatment group during the current month is compared to their cumulative activity reported the previous month, noting any significant changes in engagement for each participant. Coordinators receive specific recommendations for outreach to their study participants in order to optimize engagement, highlighting those whose engagement has notably decreased in the past month.
  2.5. The project coordinator is notified by direct message with an executive summary of any noteworthy overall trends or discrepancies. The executive summary and any action items are also shared at the next weekly START Team meeting.
  2.6. Documentation of Engagement: Past engagement reports are stored in study staff shared drive. In addition to being communicated directly, study staff have access to these shared weekly and monthly engagement reports.

## Outcomes

START’s primary outcome of interest is HIV viral load at 6 months through HemaSpot™ DBS collection kit (Table 2). Secondary outcomes of interest measured using validated scales include stimulant use (NM-ASSIST) [70]; ART adherence (Wilson) [77]; ART adherence (VAS) [78] and positive affect (modified DES) [79] (Table 2). Although reduction in sexual transmission risk is important [30], it is not the focus of the intervention; however, since it is intertwined with adherence and drug use, we will assess reduction in potentially amplified transmission risk as a secondary outcome at 6- and 12-month follow-up.

**Table 2:** Study measures and administration schedule

		Assessment					
Variable and measure	Items	BL I	BL II	3-month	6-month	9-month	12-month
**Primary Outcome**							
HIV viral load (DBS)	NA	X^a^	—^b^	—	X	—	X
**Secondary Outcomes**							
Stimulant use (NM-ASSIST) [70]	7^c^	X	—	X	X	X	X
ART adherence (Wilson) [77]	3	X	—	X	X	X	X
ART adherence (VAS) [78]	1	X	—	X	X	X	X
Positive affect (modified DES) [79]	26	X	X	X	X	X	X
**Other Variables of Interest**							
Information, motivation, and behavioral skills (LW-IMB-AAQ) [60]	18	X	—	X	X	X	X
ART adherence self-efficacy (HIV-ASES) [80]	17	X	—	X	X	X	X
Engagement in HIV care (Appendix 1)	7	X	—	X	X	X	X
Sexual behavior in aggregate (Appendix 1)	12	X	—	X	X	X	X
Sexual behavior by encounter [81]	19	X	—	—	X	—	X
Depression and anxiety (PHQ-4) [82]	4	X	—	X	X	X	X
Relationship problems (ASI Lite) [83]	9	—	X	—	X	—	X
Social support (MAPSS-SF) [84]	3	—	X	—	X	—	X
Technology use [85]	12	—	X	—	—	—	—
eHealth literacy (eHEALS) [86]	9	—	X	—	—	—	—

*Note* BL = baseline; DBS = dried blood spot; NM-ASSIST = NIDA-modified alcohol, smoking, and substance involvement screening test; ART = antiretroviral therapy; VAS = visual analog scale; DES = differential emotions scale; LW-IMB-AAQ = LifeWindows information–motivation–behavioral skills art adherence questionnaire; HIV-ASES = HIV treatment adherence self-efficacy scale; PHQ-4 = 4-item patient health questionnaire; ASI = addiction severity index; MAPPS-SF = multifactorial assessment of perceived social support, short form; eHEALS = eHealth literacy scale

^a^ X indicates measure is included in assessment

^b^ — indicates measure is not included in assessment

^c^ Items asked for each of ten substances, including methamphetamine and cocaine

### Incentives

Participants will receive incentives electronically to their email through electronic gift card code. Once participants have completed study activities, they will be paid on a bi-weekly basis by the project coordinator. E-gift card incentives will be sent to participants via email where they can redeem the value online for Visa or Mastercard prepaid cards or store-specific gift cards for over 200 different retailers. For DBS kits, the University of Miami will track weekly specimen logs by participant user ID, and the project coordinator will email the participant a gift card code. The consent form will describe the financial incentives for each study time point: $50 for each survey, at baseline, baseline 2, 3-, 6-, 9-, and 12-month follow-up, $35 for HemaSpot™ DBS collection at baseline, $70 for DBS collection at 6- and 12-month follow-up, and $25 for completing onboarding procedures during randomization for a chance to earn up to $500 in incentives.

### Statistical analyses

All study data will be interrogated using both numeric and graphical exploratory data analysis (EDA) methods. The EDA will include but not be limited to frequency tables for all categorical variables (e.g., counts and percentages) and measures of central tendency and variability for continuous variables (e.g., means, medians, standard deviations, IQR, etc.). Range checks will be defined, a priori, for every variable, and odd values will be automatically flagged and reported in nearly real-time reports. This process will allow for the immediate detection of missing data and will the study team to intervene if critical data is missing or implausible. Parametric or non-parametric tests, based on EDA findings (e.g., Chi-square tests, t-tests, Wilcoxon tests), will be used to investigate equality of potential confounders across the START and informational control conditions. If preliminary analyses detect non-trivial imbalance in potential confounders across the intervention and comparison groups that cannot be satisfactorily addressed via covariate adjustment, we will substitute causal inference methods based on the Rubin Causal Model (e.g., propensity score weighting; marginal structural models) [87–91] to obtain population-level effects of the intervention under the assumption of balanced confounders between the groups.

Based on our extensive experience working with this population and documented success with achieving 80% retention at 12 months in the RCT of ARTEMIS, we anticipate up to 20% attrition over the 12-month investigation period. All analysis methods, (i.e., mixed effects models) will be selected to use whatever data is available for each person at each time point. Attrition analyses will compare respondents who complete all measurements to those who do not based on baseline characteristics. Direct maximum likelihood and multiple imputation will be used to address incomplete data because these methods make the relatively mild assumption that missing data arise from a conditionally missing-at-random process [92].

#### Aim 1a

inferential analyses for the primary outcome, viral suppression at six months. Compared to an informational control condition, participants randomized to the START intervention will:

Hypothesis (HYP) 1a: display greater viral suppression at 6 months.

#### Aim 1b

inferential analyses for secondary outcome measures. Compared with an informational control condition, participants randomized to START will:

HYP 1b (a): Display greater viral suppression at 12 months. HYP 1b (b): Have a lower probability of amplified HIV transmission risk. HYP 1b (c): Report greater increases in ART adherence. HYP 1b (d): Report greater increases in theory-based psychological processes such as positive affect, motivation, and self-efficacy.

We plan to test these longitudinal hypotheses and will model outcomes using multilevel random coefficient models (i.e., hierarchical linear modeling; HLM). These models incorporate random intercepts and slopes for each participant based on the participant’s multiple measurements over time [93]. Initial models will follow an intent-to-treat (ITT) approach and compare outcome trajectories for participants in START intervention and informational control condition across time via the group-by-time interaction effect. A priori planned comparisons to address Hypotheses 1a–1b (d) will be performed to test group differences at 6 and 12 months. These planned comparisons will be evaluated at α = 0.05; any subsequent post-hoc comparisons will be adjusted via simulation-based stepdown methods to maintain a nominal Type 1 error rate of 0.05. Multilevel models for continuous outcomes (e.g., positive affect) will be fitted to the data using SAS PROC MIXED [94]. Multilevel models for ordered categorical or binary outcomes (e.g., probability of urine reactive urine toxicology for stimulants and sex risk) will be fitted using SAS PROC GLIMMIX with maximum likelihood estimation via adaptive quadrature with a minimum of 15 integration points [95]. Additional exploratory analyses will investigate moderators of the direct effects of the intervention on ART adherence and viral suppression. Random coefficient models to address hypotheses 1a-1b (d) will be extended to explore moderation by including moderator-by-intervention group and moderator-by-moderator product terms. Secondary analyses will also explore whether the potential mechanisms of change (e.g., reduced stimulant use) mediate the effects of START on ART adherence and viral suppression using the contrast weight-based approach of Kenny for assessing mediation in multilevel models [96]. Multilevel model assumptions (e.g., normal, homoscedastic variances) will be checked; data that do not conform to heteroskedastic-consistent estimators will be substituted for model-based estimators [97].

Secondary analyses will estimate the association between individual features of the START intervention (e.g., number of logins, days self-reported ART, number of modules completed) with ART adherence and VL suppression. These components of the intervention will be treated as predictor variables in regression models with the previously described ART adherence and VL suppression measures the dependent variables. The analytic approach for these secondary analyses will follow those for the primary aims, with regression models (linear or logistic) containing intervention arm as the primary independent variable and adjust for any necessary demographic factors.

### Power and sample size

Power analyses, using PROC POWER in SAS 9.4, were conducted to support Aim 1a Hypothesis 1 (i.e., men who are in the active START group will have greater VL suppression at 6 months compared to the control group). Treatment effects for the intervention and control groups were estimated using the observed six-month viral suppression success rates from the RCT of ARTEMIS. That is, the virus suppression rate will approximate 29/45 = 64% (80% CL: 55–73) for untreated men and 40/42 = 95% (80% CL: 89–98) for treated. To account for the uncertainty in those estimates, the pessimistic (most similar) percentage estimates from 80% Agresti-Coull confidence limits (72 vs. 89%) were used to estimate the effect for the untreated and treated groups. Beginning with 175 men per group and assuming an attrition rate of no more than 20% (effective N at six months of 140), we have approximately 90% power to detect a statistically significant difference with an alpha error rate of 5% using a two-sided Fisher’s Exact test for the two proportions at six months. This is sufficient statistical power to detect a difference in VL suppression as low as 16% between the START intervention and informational control conditions at 6 months. Even with 70% retention, power remains above 85% for detecting an effect of START on VL suppression.

### Economic evaluation

Accurate economic data is necessary for making informed decisions about implementing protocols, assessing budget impact, identifying financial or resource barriers to implementation, and ensuring fiscal sustainability over time. Our team will gather comprehensive information on the resources used and the associated costs of providing the intervention during the RCT of START. This is crucial for ensuring the success of our project and achieving our desired outcomes. Gathering economic data beforehand enables a more accurate micro-costing analysis of intervention costs and comparison with the control condition. This helps to identify any financial or resource barriers to implementation. The unique aspect of mHealth interventions is that they require a significant upfront investment to develop and launch the platform, but once established, the ongoing costs per participant are relatively low.

We will be comparing the cost of implementing START to the control conditions from the provider perspective. This analytic perspective will determine the resources that are relevant for the cost analysis, such as direct costs of service delivery for providers and reimbursement rates for payers. We will be obtaining cost information from annual budgets and financial reports, excluding any costs that are specific to research rather than clinical/monitoring activities. The goal is to accurately calculate the expenses of providing START. This will help stakeholders decide if it is possible to expand its implementation with the current resources and reimbursement methods.

To implement START, there are several expenses to consider. These include start-up/training costs, personnel expenses (including fringe benefits), contracted services, consulting services, supplies and equipment, and administrative costs. Consulting services may not apply in all situations. In our analysis, we will include the cost of sending and processing DBS samples. As part of a sensitivity analysis, we will examine how total implementation costs change if such expenditures are not factored in. The key economic data for both scenarios will include the *total yearly expenses*, the *differential costs between START and the control group*, and the *average yearly expense per participant*.

The cost data will be used to conduct a cost-effectiveness analysis (CEA) of START. This analysis will determine the additional cost required to achieve viral suppression at six and twelve months in START compared to the control group. The economic analysis will also evaluate the broader impact on the health sector by examining changes in non-study medical services utilization and costs for general medical care, HIV care, and behavioral health care. As part of the main trial outcome assessments, male participants will report their healthcare utilization services in the past six months, which will include emergency department visits, urgent care visits, inpatient hospital nights, HIV primary care appointments, and number of blood draws for HIV disease markers (CD4 + count and viral load). As part of the evaluation of START, we will systematically assess utilization of mental health and substance use disorder treatment services, such as individual and group counseling sessions, inpatient treatment nights, outpatient treatment days, and 12-step meeting attendance. We can convert counts of different types of physical and behavioral health services into dollars using monetary conversion factors. It is important to note that a visit to the emergency department can cost $2,996 on average. In contrast, a night spent in residential substance use treatment costs $177 on average. By reducing the use of healthcare services that are unnecessary, this will generate a significant reduction in costs to the health sector, which will be captured as part of the economic evaluation of START.

Finding the most cost-effective interventions can be challenging, but a CEA can be used to calculate the incremental difference in costs and outcomes between two or more alternative interventions. For instance, one can compare the cost of a new intervention to the cost of usual care and assess differences in core clinical measures such as medication adherence or retention in care. The resulting information can be used to identify the more cost-effective intervention, with a lower cost-effectiveness ratio. The analysis calculates the incremental cost-effectiveness ration to assess the additional cost per unit of desired outcome in an experimental condition relative to a control condition. We will perform sensitivity analyses to determine the impact of different cost estimates (for example, assuming that costs will decrease over time as a result of economies of scale) and outcome parameters (such as changes in the rates of viral suppression or the ability to maintain viral suppression during follow-up) on the cost-effectiveness results.

## Discusson

The collaborative, multi-site team of the START Study has learned various lessons regarding recruitment, retention, and implementation of a national remote mHealth RCT for SMM living with HIV. While our team has identified a large national sample of potentially eligible SMM with HIV who screen positive for a stimulant use disorder and report suboptimal ART adherence, only one-in-four have enrolled in the RCT. This emphasizes the immense amount of time, effort and funding needed to enroll high priority populations in clinical trials. Individuals in 43 EHE priority jurisdictions and all 50 states showed willingness to be involved in research, given ample opportunity and a non-judgmental environment.

**Fig. 2:**
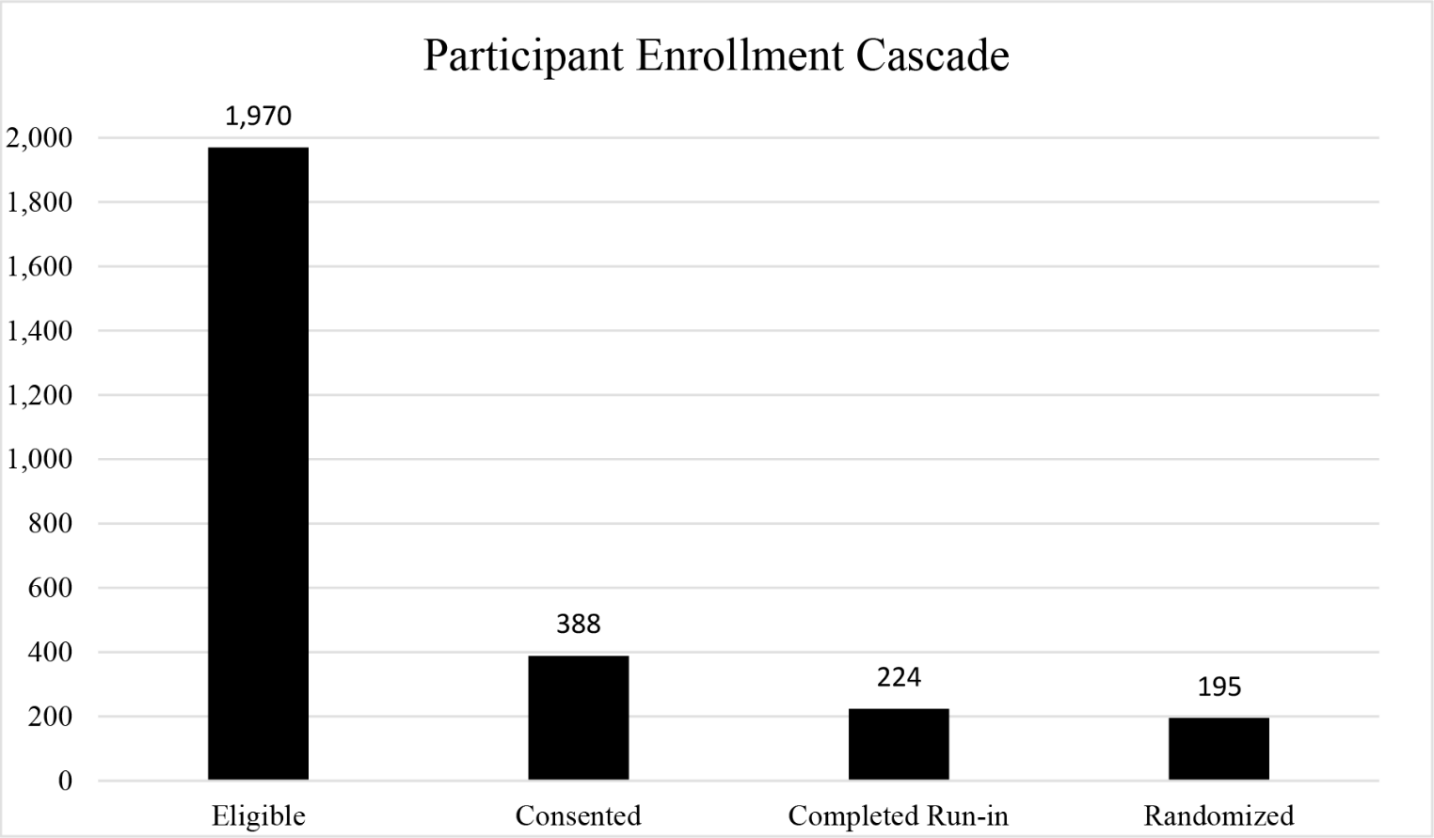
START study participant enrollment cascade June 2022-December 2023

As of December 2023, 24,515 people have taken part in our screeners to determine their study eligibility, with 1,970 (8%) eligible participants based on our enrollment criteria. With our current expenditures of $117,750 to advertise START on social networking applications and our reach of 1,970 eligible participants, our average cost per potential recruit is $59.78. This figure highlights the substantial cost of accessing SMM living with HIV who use stimulants, a hard-to-reach population. Furthermore, of those who screened eligible, 388 (19.7%) have attended an online appointment to provide informed consent and documentation that they are taking ART (Fig. 2). Our staff spends considerable time texting and recruiting eligible participants, sending study visit reminders, and rescheduling participants who miss their appointments.

Additionally, the run-in period has proven to be crucial for maintaining scientific rigor and reproducibility of this RCT, such that only half of enrolled participants complete the required study activities and attend a randomization visit. Among the 388 participants who provided informed consent, 224 (58%) have completed the run-in period and 195 (50%) have been randomized. The drop-off from consent to randomization also highlights the time, resources and over enrollment needed to meet our target randomization number. Our team devotes ample time communicating with participants to complete surveys and DBS samples. While START participants find self-collection of DBS kits overall both feasible and acceptable, we see most of our attrition resulting from loss of contact during the DBS phase of the run-in period (approximately 30%). Ensuring participants are willing and able to send viable DBS samples at baseline ensures participants who are randomized to the study arms can adhere to the RCT procedures.

**Fig. 3:**
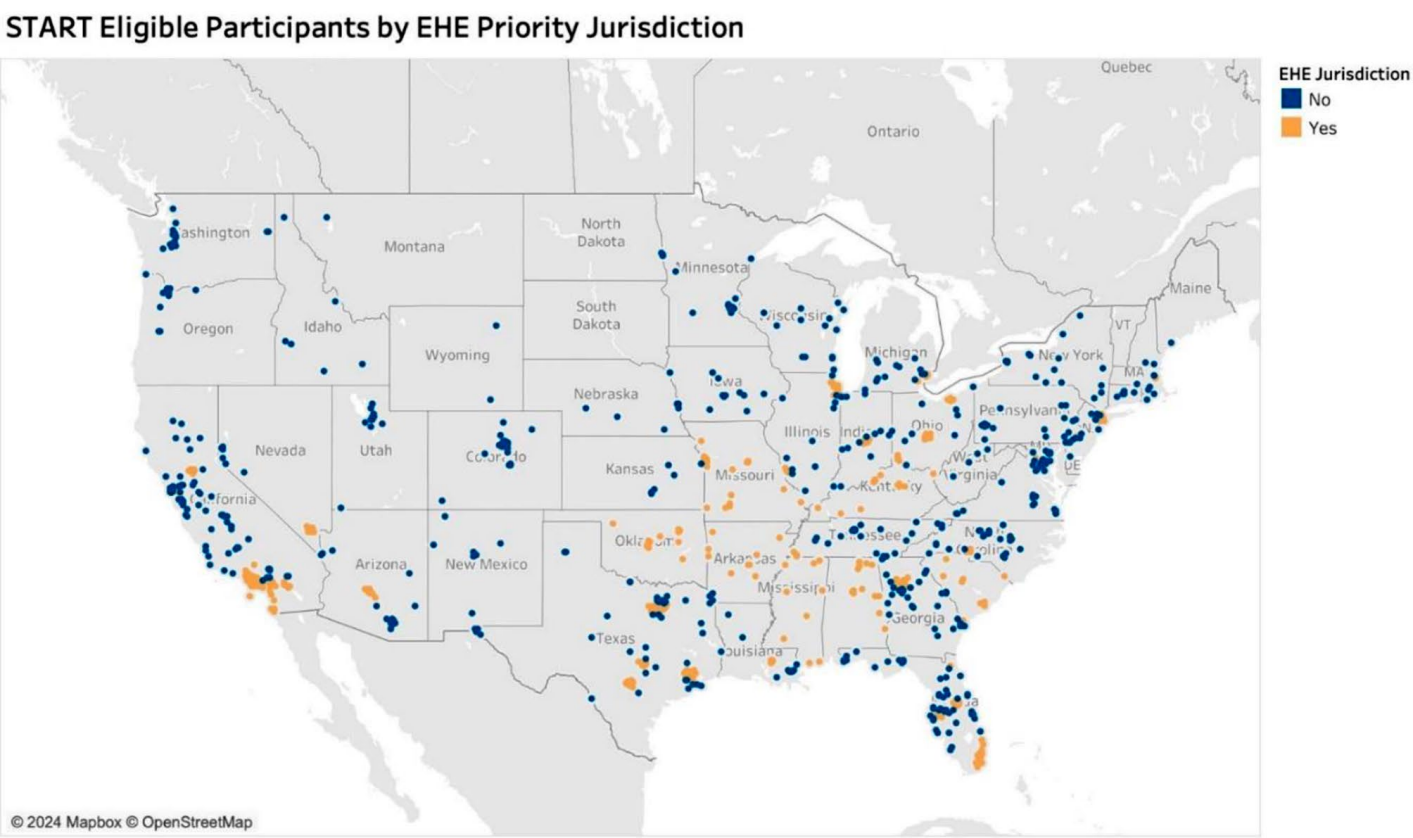
START study eligible participants by ending the epidemic plan priority jurisdiction

Of those who screened eligible for START study, approximately 44% reside in high priority regions for EHE initiative (Fig. 3) [98]. The EHE initiative uses six HIV data indicators to measure progress toward national 2025 and 2030 HIV prevention goals, including HIV incidence, knowledge of HIV status and viral load suppression. The EHE’s goals include reducing new HIV infections in the United States by 75% by 2025 and by 90% by 2030, as well as advancing health equity by scaling up key HIV prevention and treatment strategies [99]. Both goals are in alignment with START’s objective of testing an mHealth app aimed at improving medication adherence and viral load suppression, thereby reducing the chance of onward HIV transmission and improving the health of SMM living with HIV. START participants reside in 43 of the 57 EHE jurisdictions (75%). This includes participants in every EHE priority state (Alabama, Arkansas, Kentucky, Mississippi, Missouri, Oklahoma, South Carolina) [99]. START participants also reside in 36 EHE priority counties, including Miami-Dade County, Florida; Wayne County, Michigan; Suffolk County, Massachusetts; Fulton County, Georgia and LA County, California.

Overall, our findings will guide adequate resource allocation to achieve randomization targets in future mHealth research among SMM living with HIV who use stimulants. Significant resources in terms of intervention development and implementation, recruitment costs and staff time are needed to engage SMM living with HIV who screen positive for stimulant use disorders. Future mHealth behavioral interventions will benefit from this work and further support the EHE goals of advancing health equity through scalable HIV treatment interventions in the era of TasP.

### Electronic supplementary material

Below is the link to the electronic supplementary material.

Supplementary Material 1

## Data Availability

The datasets used and/or analyzed during the current study are available from the corresponding author on reasonable request.

## Abbreviations

ART: Anti-retroviral therapy
APP+: ART adherence application
CEA: Cost-effectiveness analysis
CM: Contingency management
DBS: Dried blood spot
DES: Modified Differential Emotions Scales
EDA: Exploratory data analysis
EHE: Ending the HIV Epidemic Plan
HLM: Hierarchical linear modeling
HYP: Hypothesis
IMB: Information-Motivation-Behavioral Skills Model
NM: ASSIST-NIDA Modified Alcohol, Smoking, and Substance Involvement Screening Test
RCT: Randomized control trial
SMM: Sexual minority men
SOP: Standard operating procedures
START: Supporting Treatment Adherence for Resilience and Thriving study
TasP: Treatment as prevention
VAS: ART adherence visual analog scale
VL: Viral load
