# Supplementary material for: Supporting treatment adherence for resilience and thriving (START): protocol for a mHealth randomized controlled trial
Source: BMC Public Health. 2024 Aug 29;24:2350. doi: 10.1186/s12889-024-19745-7 (PMC11360769; doi:10.1186/s12889-024-19745-7)
Supplement: Supplementary file 1 — Supplementary Material 1 [file 12889_2024_19745_MOESM1_ESM.docx]

APPENDIX 1- ADDITIONAL START MEASURES FROM TEAM

Start of Block: Engagement in HIV Care (ENG)

ENG_0A The following questions are about health care related to HIV in the past 6 months.

| 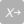 |
| --- |

ENG1_0A In the past 6 months, did you schedule any medical appointments with an HIV primary care provider (e.g., a physician, physician’s assistant, or nurse practitioner)?

- Yes (1)
- No (0)
- Decline to answer (999)

Skip To: ENG4_0A If In the past 6 months, did you schedule any medical appointments with an HIV primary care provider... = No

| 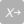 |
| --- |

ENG2_0A In the past 6 months, how many medical appointments did you schedule with your HIV primary care provider (e.g., a physician, physician’s assistant, or nurse practitioner)?

- Number of appointments: (1) __________________________________________________
- Decline to answer (999)

Display This Question:

If If In the past 6 months, how many medical appointments did you schedule with your HIV primary care p... Text Response Is Greater Than or Equal to 1

| 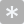 | 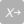 |
| --- | --- |

ENG3_0A Of these ${ENG2_0A/ChoiceTextEntryValue/1} HIV primary care appointments that you scheduled, how many did you…

|  | Number of appointments (1) |
| --- | --- |
| attend? (ENG3A_0A) |  |
| miss? (ENG3B_0A) |  |
| Total |  |

| 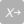 |
| --- |

ENG4_0A In the past 6 months, did you have blood drawn to measure your HIV viral load?

- Yes (1)
- No (0)
- Decline to answer (999)

| 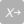 |
| --- |

ENG5_0A What were the results of your most recent viral load test?

- Detectable (1)
- Undetectable (0)
- Not sure / don't remember (888)
- Decline to answer (999)

| 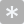 | 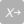 |
| --- | --- |

ENG6_0A When did you first start taking HIV medications?

|  | Don't know (888) | Decline to answer (999) |
| --- | --- | --- |
| Month (MM) (ENG6A_0A) |  |  |
| Year (YYYY) (ENG6B_0A) |  |  |

Display This Question:

If When did you first start taking HIV medications? [ Don't know] (Count) >= 1

| 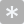 | 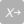 |
| --- | --- |

ENG7_0A Your answer is really important to us. Please make your best guess: when did you first start taking HIV medications?

|  | Decline to answer (999) |
| --- | --- |
| Month (MM) (ENG7A_0A) |  |
| Year (YYYY) (ENG7B_0A) |  |

| 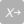 |
| --- |

ENG8_0A In the past 6 months, did an HIV primary care provider tell you to stop taking your HIV medications?

- Yes (1)
- No (0)
- Decline to answer (999)

End of Block: Engagement in HIV Care (ENG)

Start of Block: Sexual Risk in Aggregate & by Encounter (RISK)

RISK0_0A How old were you the first time you had anal sex with another male?

- Younger than 10 (0)
- 10 (1)
- 11 (2)
- 12 (3)
- 13 (4)
- 14 (5)
- 15 (6)
- 16 (7)
- 17 (8)
- 18 (9)
- 19 (10)
- 20 or older (11)
- Don’t know or not sure (777)
- Decline to answer (999)

| 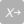 |
| --- |

RISK1_0A How old was he?

- Younger than 10 (1)
- 10 (1)
- 11 (2)
- 12 (3)
- 13 (4)
- 14 (5)
- 15 (6)
- 16 (7)
- 17 (8)
- 18 (9)
- 19 (10)
- 20 or older (11)
- Don’t know or not sure (777)
- Decline to answer (999)

RISK_0A This section asks about different types of anal sex with men in the **last 3 months**. If you cannot give exact numbers, please give your best guess for each type of anal sex.

| 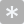 |
| --- |

RISK2_0A During the last 3 months, with how many **men** have you had **anal sex**?

________________________________________________________________

Skip To: End of Block If Condition: During the last 3 months, w... Is Equal to 0. Skip To: End of Block.

| 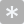 |
| --- |

RISK3_0A During the last 3 months, with how many **men** have you had **receptive anal sex**  (you were the bottom)?

________________________________________________________________

Skip To: RISK4_0A If Condition: This section asks about dif... Is Equal to 0. Skip To: In the last 3 months, with how many m....

| 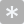 |
| --- |

RISK3A_0A Of these ${RISK3_0A/ChoiceTextEntryValue} men with whom you had receptive anal sex (you were the bottom), how many were **without a condom**?

________________________________________________________________

Skip To: RISK4_0A If Condition: Of these (SB1) men with who... Is Equal to 0. Skip To: In the last 3 months, with how many m....

| 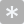 |
| --- |

RISK3B_0A Of these ${RISK3A_0A/ChoiceTextEntryValue} men with whom you had receptive anal sex (you were the bottom) **without a condom**, how many were taking PrEP?

________________________________________________________________

| 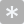 |
| --- |

RISK3C_0A Of these ${RISK3A_0A/ChoiceTextEntryValue} men with whom you had receptive anal sex (you were the bottom) **without a condom**, how many told you they were taking PrEP?

________________________________________________________________

| 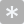 |
| --- |

RISK3D_0A Of these ${RISK3A_0A/ChoiceTextEntryValue} men with whom you had receptive anal sex (you were the bottom) **without a condom**, how many did you guess were taking PrEP?

________________________________________________________________

| 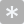 |
| --- |

RISK3E_0A Of these ${RISK3A_0A/ChoiceTextEntryValue} men with whom you had receptive anal sex (you were the bottom) **without a condom**, how many were living with HIV?

| 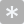 |
| --- |

RISK4_0A In the last 3 months, with how many men did you have **insertive anal sex** (you were the top)?

________________________________________________________________

Skip To: RISK_S_0A If Condition: In the last 3 months, with ... Is Equal to 0. Skip To: Please tell us about the last man you....

| 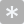 |
| --- |

RISK4A_0A Of these ${RISK4_0A/ChoiceTextEntryValue} men with whom you had insertive anal sex (you were the top), how many were **without a condom**?

________________________________________________________________

Skip To: RISK_S_0A If Condition: Of these ${q://QID103/... Is Equal to 0. Skip To: Please tell us about the last man you....

| 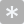 |
| --- |

RISK4B_0A Of these ${RISK4A_0A/ChoiceTextEntryValue} men with whom you had insertive anal sex (you were the top) **without a condom**, how many were taking PrEP?

________________________________________________________________

| 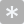 |
| --- |

RISK4C_0A Of these ${RISK4A_0A/ChoiceTextEntryValue} men with whom you had insertive anal sex (you were the top) **without a condom**, how many told you they were taking PrEP?

________________________________________________________________

| 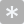 |
| --- |

RISK4D_0A Of these ${RISK4A_0A/ChoiceTextEntryValue} men with whom you had insertive anal sex (you were the top) **without a condom**, how many did you guess were taking PrEP?

________________________________________________________________

| 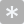 |
| --- |

RISK4E_0A Of these ${RISK4A_0A/ChoiceTextEntryValue} men with whom you had insertive anal sex (you were the top) **without a condom**, how many were living with HIV?

RISK_S_0A Please tell us about the last man you had anal sex with in a one-on-one encounter.             The last man I had anal sex with was (*enter a nickname or initials to help you remember*):

RISK5_0A Which of the following best describes your relationship with ${RISK_S_0A/ChoiceTextEntryValue}?

- Main sex partner (someone you feel committed to above all others) (1)
- Casual sex partner (someone you've sex with multiple times who is not your main partner) (2)
- One-time sex partner (someone you had sex with only once who is not your main partner) (3)
- Decline to answer (999)

| 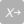 |
| --- |

RISK6_0A Is ${RISK_S_0A/ChoiceTextEntryValue} someone you had sex with in exchange for money, food, or drugs?

- No (0)
- Yes, I received money, food, or drugs (1)
- Yes, I provided money, food, or drugs (2)
- Decline to answer (999)

| 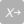 |
| --- |

RISK7_0A Which of the following best describe ${RISK_S_0A/ChoiceTextEntryValue}’s racial/ethnic identity? Select all that apply.

- Asian/Asian American (1)
- Black/African American/Caribbean Black/African Black/multiethnic Black (2)
- Hispanic/Latino (3)
- Native American or Alaskan Native (4)
- Native Hawaiian or other Pacific Islander (5)
- White (6)
- Other (7)
- Don’t know (888)
- Decline to answer (999)

| 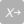 |
| --- |

RISK8_0A Is ${RISK_S_0A/ChoiceTextEntryValue} younger, older, or about the same age as you?

- Younger (1)
- Older (2)
- About the same age (3)
- Don’t know (888)
- Decline to answer (999)

| 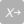 |
| --- |

RISK9_0A The last time you had sex with ${RISK_S_0A/ChoiceTextEntryValue}, did you know his HIV status?

- Yes, positive (1)
- Yes, negative (2)
- No, I assume positive (3)
- No, I assume negative (4)
- I didn’t think about it (5)
- Decline to answer (999)

Display This Question:

If The last time you had sex with ${q://QID118/ChoiceTextEntryValue}, did you know his HIV status? = Yes, positive

Or The last time you had sex with ${q://QID118/ChoiceTextEntryValue}, did you know his HIV status? = Yes, negative

| 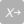 |
| --- |

RISK9A_0A How did you know?

- I asked him first (1)
- He told me first (2)
- He told me after I told him my status (3)
- Someone else told me (4)
- I saw his HIV status on his dating profile (online/app) (5)
- Other (6)
- Don’t remember (888)
- Decline to answer (999)

Display This Question:

If The last time you had sex with ${q://QID118/ChoiceTextEntryValue}, did you know his HIV status? = Yes, positive

| 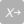 |
| --- |

RISK9B_0A If ${RISK_S_0A/ChoiceTextEntryValue} is HIV-positive, was he prescribed HIV medication?

- No, he said he was not taking HIV medication (0)
- Yes, he said he was taking HIV medication (1)
- I don’t know (we didn’t talk about it) (2)
- Decline to Answer (999)

Display This Question:

If The last time you had sex with ${q://QID118/ChoiceTextEntryValue}, did you know his HIV status? = Yes, negative

| 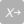 |
| --- |

RISK9C_0A If ${RISK_S_0A/ChoiceTextEntryValue} is HIV-negative, was he taking PrEP (pre-exposure prophylaxis)?

- No, he said he was not on PrEP (0)
- Yes, he said he was on PrEP (1)
- I don’t know (we didn’t talk about it) (2)
- Decline to answer (999)

| 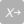 |
| --- |

RISK10_0A The last time you had sex with ${RISK_S_0A/ChoiceTextEntryValue}, did he know you were HIV positive?

- No (0)
- Yes (1)
- Don’t know (888)
- Decline to answer (999)

Display This Question:

If The last time you had sex with ${q://QID118/ChoiceTextEntryValue}, did he know you were HIV posit... = Yes

| 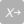 |
| --- |

RISK10A_0A How did he know?

- He asked me first (1)
- I told him first (2)
- I told him after he told me his status (3)
- Someone else told him (4)
- My HIV status is on my dating profile (online/app) (5)
- Other (6)
- I don’t remember (888)
- Decline to answer (999)

Display This Question:

If The last time you had sex with ${q://QID118/ChoiceTextEntryValue}, did he know you were HIV posit... = Yes

| 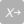 |
| --- |

RISK10U_0A Did you talk about your viral load status?

- Yes, I told him I was detectable (1)
- Yes, I told him I was undetectable (2)
- No, we didn’t talk about it (0)
- Decline to answer (999)

| 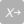 |
| --- |

RISK11_0A The last time you had sex with ${RISK_S_0A/ChoiceTextEntryValue}, did you use stimulants within 2 hours before or during sex?

- No (0)
- Yes (1)
- Decline to answer (999)

Display This Question:

If The last time you had sex with ${q://QID118/ChoiceTextEntryValue}, did you use stimulants within... = Yes

| 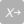 |
| --- |

RISK11A_0A How did you use stimulants? (Check all that apply.)

- Smoked (1)
- Snorted (2)
- Hotrail (3)
- Booty bump (4)
- Injected (5)
- Decline to answer (999)

| 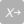 |
| --- |

RISK12_0A Did ${RISK_S_0A/ChoiceTextEntryValue} use stimulants within 2 hours before or during sex?

- No (0)
- Yes (1)
- Decline to answer (999)

Display This Question:

If Did ${q://QID118/ChoiceTextEntryValue} use stimulants within 2 hours before or during sex? = Yes

|  |
| --- |

RISK12A_0A How did he use stimulants? (Check all that apply.)

- Smoked (1)
- Snorted (2)
- Hotrail (3)
- Booty bump (4)
- Injected (5)
- Decline to Answer (999)

RISK13_0A The last time you had sex with ${RISK_S_0A/ChoiceTextEntryValue}, did he have trouble with getting or keeping an erection?

- No (0)
- Yes (1)
- Decline to answer (999)

Display This Question:

If The last time you had sex with ${q://QID118/ChoiceTextEntryValue}, did he have trouble with getti... = Yes

| 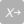 |
| --- |

RISK13A_0A Did he take medication (e.g., Viagra) to get or keep an erection?

- No (0)
- Yes (1)
- Decline to answer (999)

| 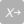 |
| --- |

RISK14_0A When you last had sex with ${RISK_S_0A/ChoiceTextEntryValue}…   Was his penis in your mouth?

- No (0)
- Yes (1)
- Decline to answer (999)

Display This Question:

If When you last had sex with ${q://QID118/ChoiceTextEntryValue}…   Was his penis in your mouth? = Yes

| 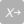 |
| --- |

RISK14A_0A Did he ejaculate (cum) in your mouth?

- No (0)
- Yes (1)
- Decline to answer (999)

| 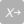 |
| --- |

RISK15_0A Was his penis in your anus?

- No (0)
- Yes (1)
- Decline to answer (999)

Display This Question:

If Was his penis in your anus? = Yes

| 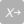 |
| --- |

RISK15A_0A While his penis was in your anus…
Did he wear a condom?

- No (0)
- Yes (1)
- Not sure (2)
- Decline to answer (999)

Display This Question:

If Was his penis in your anus? = Yes

| 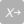 |
| --- |

RISK15B_0A Did he ejaculate (cum)?

- No (0)
- Yes (1)
- Not sure (2)
- Decline to answer (999)

| 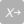 |
| --- |

RISK16_0A The last time you had sex with ${RISK_S_0A/ChoiceTextEntryValue}, did you have trouble with getting or keeping an erection?

- No (0)
- Yes (1)
- Decline to answer (999)

Display This Question:

If The last time you had sex with ${q://QID118/ChoiceTextEntryValue}, did you have trouble with gett... = Yes

| 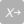 |
| --- |

RISK17_0A Did you take medication (e.g., Viagra) to get or keep an erection?

- No (0)
- Yes (1)
- Decline to answer (999)

| 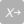 |
| --- |

RISK18_0A When you last had sex with ${RISK_S_0A/ChoiceTextEntryValue}… 


Was your penis in his mouth?

- No (0)
- Yes (1)
- Decline to Answer (999)

Display This Question:

If When you last had sex with ${q://QID118/ChoiceTextEntryValue}…  Was your penis in his mouth? = Yes

| 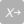 |
| --- |

RISK18A_0A Did you ejaculate (cum) in his mouth?

- No (0)
- Yes (1)
- Decline to answer (999)

| 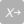 |
| --- |

RISK19_0A Was your penis in ${RISK_S_0A/ChoiceTextEntryValue}’s anus?

- No (0)
- Yes (1)
- Decline to answer (999)

Display This Question:

If Was your penis in ${q://QID118/ChoiceTextEntryValue}’s anus? = Yes

| 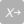 |
| --- |

RISK19A_0A While your penis was in his anus…
 Did you wear a condom?

- No (0)
- Yes (1)
- Not sure (2)
- Decline to answer (999)

Display This Question:

If Was your penis in ${q://QID118/ChoiceTextEntryValue}’s anus? = Yes

| 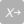 |
| --- |

RISK19B_0A Did you ejaculate (cum)?

- No (0)
- Yes (1)
- Not sure (2)
- Decline to answer (999)

| 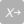 |
| --- |

RISK19C_0A How enjoyable was the sex?

- Extremely Unenjoyable (0)
- Unenjoyable (1)
- Enjoyable (2)
- Extremely Enjoyable (3)
- Decline to answer (999)

End of Block: Sexual Risk in Aggregate & by Encounter (RISK)
